# Nineteenth Annual Meeting of the Central New York Medical Society

**Published:** 1886-07

**Authors:** Edward B. Angell

**Affiliations:** Rochester, N. Y.


### Society Reports.

---

### Nineteenth Annual Meeting of the Central New York Medical Society.

Reported by Edward B. Angell, M. D., Rochester, N. Y.

The Medical Society of Central New York held its annual meeting this month in Rochester. There was a large attendance. Dr. John O. Roe presided, and Dr. J. H. Arnold, of

Clyde, acted as secretary. Delegates were present from Onondaga, Cayuga, Seneca, Ontario, Wayne, Monroe and Steuben counties. Dr. Roe delivered the president's annual address, taking as his subject, "Differentiation in Medicine, the Necessary Result of the Law of Evolution." He made a general survey of the history of medicine from the earliest period, and formulated the following propositions:

*First.*—It is an undeniable or self-evident fact, that "at the present day medical science has expanded to such an extent that its intelligent cultivation as a whole, by one person, has become impossible."

*Second.*—That this great expansion in medicine has come about by the intelligent devotion of individuals, mainly or wholly, to the cultivation of one department or branch.

*Third.*—That the practical and natural outcome of this individual devotion, mainly or wholly, to one branch or department has correspondingly increased, or better fitted the individual to apply this knowledge to the cure of disease.

*Fourth.*—That true conservative, scientific specialism in the practice of medicine, is but a healthful and necessary outgrowth from this intelligent study and cultivation of the different departments.

Medical specialism may be divided into two classes:

*First.*—The empyrical specialist, the one who arrogates to himself special knowledge concerning the treatment of certain diseases which has been acquired only by himself, or who has attained a dexterity in certain manipulations or operations that none others possess, or one who, without any special fondness or fitness by previous training, adopts a speciality, as a supposed easy and short road to fame and fortune.

*Second.*—The rational or true specialist, the one who, while recognizing the impossibility of cultivating equally well every department of medicine, directs the resources he has gathered from the whole field of medicine to the enriching of one department.

A specialist in the highest sense may well be defined as "one who knows something of everything, and everything of something." Dr. George Johnson, of London, says: "Of the specialist, it should be said with truth, that he is not one who knows more of the particular class of diseases to which he devotes most time and especial attention and study.

To fit one, therefore, for the study of a speciality, nothing is of greater importance than a broad general culture, a thorough medical education, and a somewhat extended experience in the general practice of medicine. He must not only know all about the department to which he gives his special attention, but he must know every condition of the general system that has an influence on this part, and also every influence which a disease of this part can have on the system in general.

It is as impossible in medicine, as in all other departments of science, for one to acquire knowledge and experience so evenly that he is not relatively stronger and more competent in one direction than another. But in whatever capacity or direction we labor, whether as general practitioners or devotees to a particular branch, let us remember that all are co-workers in one common cause. Let us not be content with results however excellent, or attainments however high, but let us all work together and contribute what is in our power to lay deeper the foundations, and to build higher the walls of that noble edifice, *Medicine.*

Dr. Armanda S. Hickey, of Auburn, read a report of two cases of abdominal tumor. One, a carcinoma of the omentum; the other, a multilocular ovarian cyst. Particular attention was called to the decisive character of the information gained by a careful analysis of the fluids obtained by preliminary tapping.

In the first instance, a case of malignant growth, involving the omentum and peritoneum. The general significance of the symptoms indicated an ovarian cyst. But on tapping the abdomen, twenty pints of a greenish-yellow fluid were removed, and on palpation, a hard, irregular mass was readily made out. This mass was immovable, and, by its attachments, had prevented the intestines from floating upward, thus suggesting the idea of a cyst. An analysis of the fluid withdrawn, however, established the suspicion of malignant growth. Its sp. gr. was 1010; re-action neutral; albumen sufficient to form a solid mass on application of heat. There was also a peculiar fibrous substance, hour-glass in shape, floating in the fluid, evidently fibrine, formed shortly after the removal of the liquid. The microscope showed epithelial cells and blood corpuscles, but no granular cells.

The patient's condition was but slightly improved, and, a month later, seventeen pints more of fluid were removed. Analysis resulted as follows:

Sp. gr. 1016; re-action, neutral; color, greenish-yellow; albumen as before; fibrous strings appearing on cooling. The microscope gave only broken-down epithelium. The disease, however, progressed steadily, and death followed a month later. Examination of the abdomen sustained the diagnosis of malignant growth.

In the second case, one plainly of ovarian cyst, after preparatory treatment, an incision three and a half inches in length was made in the abdominal wall, and a multilocular ovarian cyst, weighing seventeen pounds, was removed. The progress of the patient, after the operation, was most satisfactory. The point of interest, however, was the analysis of the fluid removed, and its comparison with that of the previous case.

Its sp. gr. varied from 1010 in the largest cavity, to 1030 in some of the smaller; re-action, neural; color, clear straw; no odor; no deposit; albumen, a solid coagulum with heat; no appearance of fibrine on standing.

The microscope showed abundant transparent granular cells, varying in size from 1-1000 to 1-4000 of an inch in diameter. The two fluids were similar in sp. gr., and in the presence of albumen, but differed in two particulars of great importance in the diagnosis; the one containing fibrine, but no granular cells; the second showing the reverse of these conditions; thus establishing the widely-different pathological nature of the two growths, the external signs of which were so very similar.

Dr. Charles H. Richmond, of Lavonia, read a paper giving the results of the use of arbor vitæ in the treatment of epithelioma. In a series of several cases, he had found it of great service, and considered it worthy of more extended trial.

Dr. J. H. Jewett, of Canandaigua, read the report of a case of pneumonia complicating the ninth month of pregnancy.

On the night of March 8th, of this year, he had been called to a case of pneumonia in a woman who was expecting confinement in two weeks. She had been ill for three days, was very weak, had a high temperature, with rapid pulse, and quick, shallow respiration. Two grs. quinine, and five minims of tr. digitatis were ordered every three hours, with opium, to give relief from pain, while a mustard poultice was applied to chest, and milk given very freely. That night she was very comfortable. Next morning, after a rectal enema of water, labor was precipitated, and a healthy child, weighing nine pounds, was born, with almost entire freedom from pain, though three previous labors had been exceedingly tedious and painful, forceps being required in two instances. There was marked but temporary improvement in the febrile symptoms, the pulse dropping to 128, the respiration to 30, and the temperature to normal. But a few hours later, there was a renewal of the febrile action, with marked extension of the malady, with fatal result on the tenth day of the disease.

The case was considered of importance—

*First.*—Because of its rare occurrence at so late a period in pregnancy.

*Second.*—Because of the unusual fact of painless delivery, and the birth of a healthy child, after exposure for four days to high temperature and marked dyspnœa.

*Third.*—The question suggests itself whether, under these circumstances, labor was provoked by the accumulation of carbonic acid in the maternal circulation, as many authorities believe; or whether, as the writer himself is inclined to think, it was in consequence of the ecbolic action of the quinine?

Dr. W. S. Ely, of Rochester, read a paper entitled "The Patient her own Anæsthetizer in Natural Labor."

After referring to ether as an anæsthetic in many cases of natural labor, if used judiciously at the proper moment, and its risk, if employed indiscriminately at all stages of the delivery, he said: "I, therefore, gradually came to the adoption of a plan which practically, with some exceptions, limited the use of the anæsthetic in natural labor to the period occupied by the delivery of the child. Amid the mass of literature upon the prevention of laceration of the perineum, I recognize but one principle of value, viz.: to retard the delivery of the child as long as possible. In numerous patients, especially among the higher classes, where pelvic capacity tends to reduction, and fœtal head to disproportionate size, great care is necessary in the delivery, to avoid injury to the mother. Abundant experience has convinced me that, in the latter class of patients, the physican can be of great aid in preventing perineal lacerations, by the proper use of anæsthetics, and by retardation of delivery. In order that I might have the patient more perfectly under control, I have made her her own anæsthetizer. The procedure is as follows:

I have folded a large handkerchief in a cravat, two inches wide, which is spread on the patient's palm, carried over the back of the hand and tied around the wrist. On this ether is poured, and the patient herself covers her nose and mouth with it. When she feels a pain, she holds out the hand for the ether, the palm upwards, and from the bottle, held in the free hand of the physician, one or more teaspoonfuls is poured. When it is exhausted she asks for more, and the moment she becomes fully under its influence, the active muscular effort, necessary to hold the hand to the face, relaxes, and the hand drops. She seldom therefore, takes ether to an extent which abolishes the entire consciousness of what is going on. If this is the case, it is only momentary in duration, and the patient can be made to appreciate the peremptory command given to her to avoid any voluntary expulsive efforts when the head distends the outlet. Thus, the time of delivery is somewhat prolonged, the head extended under the action of uterine contractions alone, while the patient, under the influence of ether, to an extent which abolishes pain, is not so profoundly unconscious as to lose the power of responding to the command of the physician to desist from all auxiliary, muscular effort. He would not maintain the anæsthetic at the time when the head begins to emerge at the vulva, for uterine contraction will then be supplemented by action of the voluntary muscles, and this auxiliary expulsive force cannot be controlled if the patient is fully anæsthetized. A similar management is required for the passage of the shoulders which often are responsible for an extensive laceration.

Since the adoption of this treatment, he has had fewer and lighter perineal lacerations—a result attributed to the method described.

Dr A. W. Streeter, of Rochester, presented a paper entitled, "Fundamental Gynæcological Pathology," in which he made an earnest plea for a broader interpretation of uterine disease, and for a more comprehensive plan of treatment. Much of the inefficiency of our treatment is due to the mistake of combating symptoms only, rather than the underlying pathological condition which may express itself in a varied manner. We must not forget the anatomical texture of the pelvic region, the rich blood supply, the large amount of spongy cellular tissue, thus affording ready facilities for inflammatory extension by continuity, or contiguity of structure.

Inflammation of the mucous tracts is not confined to those surfaces with mathematical precision, but must affect as well the underlying structures.

Leucorrhœa—that affection in the treatment of which so much effort has been misdirected—is rather the expression of congestion, whether from inflammatory action, from obstructed circulation, due to pressure of whatever nature, or, again, from the relaxed or over-dilated blood-vessels of general anæmia.

A lacerated cervix, unless it interferes with the pelvic circulation, will cause little disturbance, and operative measures are uncalled for.

So with uterine displacements. Whatever the determining cause, back of all must be the pelvic congestion, and unless that be relieved, our remedial measures must fail in great part at least. In conclusion, the writer quoted at length from Dr. Emmet, who so strongly calls attention to the need of appreciating the important part played by the connective tissue structures in all uterine maladies, and the necessity of recognizing the pathological changes in the pelvic cellular tissue, as the immediate cause of the results we now regard as the original disease in the uterus and ovaries.

Dr. Charles E. Darrow, of Rochester, exhibited a self-retaining Sims speculum of his own device, which, in his work, has proved to be admirably adapted to its purposes.

It differs from other contrivances of a similar character, in being simple and entirely reliable. It has no straps, and absolutely accomplishes the work of the original instrument without any assistance.

A speculum and a saddle comprise the whole instrument. The speculum is an ordinary Sims blade, to which has been added a large upper and a small lower flange. Dr. Munde, of New York, first proposed a somewhat similar flange, with the object of raising the superior buttock of the patient, thus allowing a full field of view.

The saddle of the speculum is flattened upon two sides, and has a rapid thread cut upon the remaining two sides. It is made of a sheet of brass firm enough to hold any curve given it, yet flexible enough to admit of accommodating the form of any patient. It is lined with a sheet of corrugated, soft rubber, rendering the hold comfortable, yet tenacious.

The speculum is as readily applied as the ordinary bivalve, gives all required variations of position, and does not violate any principle of the Sims posture. It is self-retaining under all conditions of the perineum, whether ruptured or not, while its absolute security and perfect steadiness give it a decided advantage over any instrument held by the hand of an assistant.

Dr. Edward B. Angell, of Rochester, read the report of a case of dorsal myelitis accompanying pregnancy, and attended with marked hysterical phenomena. The paper will appear in full in a subsequent number.

Dr. Bailey, of Albion, read an interesting paper on Graves disease, which will appear in full in the next number of the Journal.

Other papers were read as follows: Dr. W. J. Herriman, on "Polypharmacy;" Dr. Lattimore, on "The Determination of Urea by a New Form of Apparatus;" Dr. Damis, on "Progressive Muscular Atrophy;" Dr. L. A. Weigel, of Rochester, on "Restoration of Crippled Joints." Extracts from each of the above will appear in our next number.

After the reading of the papers, the Society elected the following named officers for the ensuing year: President, Dr. J. P. Creveling, Auburn; first vice-president, Dr. A. Dann, Rochester; second vice-president, Dr. Gregory Doyle, Syracuse; secretary, Dr. J. N. Arnold, Clyde; treasurer, Dr. Alfred Mercer, Syracuse.

